# Malaria hospitalisation in East Africa: age, phenotype and transmission intensity

**DOI:** 10.1186/s12916-021-02224-w

**Published:** 2022-01-27

**Authors:** Alice Kamau, Robert S. Paton, Samuel Akech, Arthur Mpimbaza, Cynthia Khazenzi, Morris Ogero, Eda Mumo, Victor A. Alegana, Ambrose Agweyu, Neema Mturi, Shebe Mohammed, Godfrey Bigogo, Allan Audi, James Kapisi, Asadu Sserwanga, Jane F. Namuganga, Simon Kariuki, Nancy A. Otieno, Bryan O. Nyawanda, Ally Olotu, Nahya Salim, Thabit Athuman, Salim Abdulla, Amina F. Mohamed, George Mtove, Hugh Reyburn, Sunetra Gupta, José Lourenço, Philip Bejon, Robert W. Snow

**Affiliations:** 1 Kenya Medical Research Institute (KEMRI) - Wellcome Trust Research Programme, Nairobi, Kenya; 2 Department of Zoology, University of Oxford, Oxford, UK; 3 Child Health and Development Centre, College of Health Sciences, Makerere University, Kampala, Uganda; 4 Kenya Medical Research Institute (KEMRI) - Wellcome Trust Research Programme, Kilifi, Kenya; 5 Kenya Medical Research Institute (KEMRI), Centre for Global Health Research, Kisumu, Kenya; 6 Infectious Diseases Research Collaboration, Kampala, Uganda; 7 Ifakara Health Institute, Bagamoyo, Tanzania; 8 Kilimanjaro Christian Medical Centre/Joint Malaria Programme, Moshi, Tanzania; 9 London School of Hygiene and Tropical Medicine, London, UK; 10 National Institute for Medical Research, Amani Research Centre, Muheza, Tanzania; 11 Centre for Tropical Medicine and Global Health, Nuffield Department of Clinical Medicine, University of Oxford, Oxford, UK

**Keywords:** Malaria, Age pattern, Parasite prevalence, Severe malaria, Anaemia, Cerebral malaria

## Abstract

**Background:**

Understanding the age patterns of disease is necessary to target interventions to maximise cost-effective impact. New malaria chemoprevention and vaccine initiatives target young children attending routine immunisation services. Here we explore the relationships between age and severity of malaria hospitalisation versus malaria transmission intensity.

**Methods:**

Clinical data from 21 surveillance hospitals in East Africa were reviewed. Malaria admissions aged 1 month to 14 years from discrete administrative areas since 2006 were identified. Each site-time period was matched to a model estimated community-based age-corrected parasite prevalence to provide predictions of prevalence in childhood (*Pf*PR_2–10_). Admission with all-cause malaria, severe malaria anaemia (SMA), respiratory distress (RD) and cerebral malaria (CM) were analysed as means and predicted probabilities from Bayesian generalised mixed models.

**Results:**

52,684 malaria admissions aged 1 month to 14 years were described at 21 hospitals from 49 site-time locations where *Pf*PR_2–10_ varied from < 1 to 48.7%. Twelve site-time periods were described as low transmission (*Pf*PR_2–10_ < 5%), five low-moderate transmission (*Pf*PR_2–10_ 5–9%), 20 moderate transmission (*Pf*PR_2–10_ 10–29%) and 12 high transmission (*Pf*PR_2–10_ ≥ 30%). The majority of malaria admissions were below 5 years of age (69–85%) and rare among children aged 10–14 years (0.7–5.4%) across all transmission settings. The mean age of all-cause malaria hospitalisation was 49.5 months (95% *CI* 45.1, 55.4) under low transmission compared with 34.1 months (95% *CI* 30.4, 38.3) at high transmission, with similar trends for each severe malaria phenotype. CM presented among older children at a mean of 48.7 months compared with 39.0 months and 33.7 months for SMA and RD, respectively. In moderate and high transmission settings, 34% and 42% of the children were aged between 2 and 23 months and so within the age range targeted by chemoprevention or vaccines.

**Conclusions:**

Targeting chemoprevention or vaccination programmes to areas where community-based parasite prevalence is ≥10% is likely to match the age ranges covered by interventions (e.g. intermittent presumptive treatment in infancy to children aged 2–23 months and current vaccine age eligibility and duration of efficacy) and the age ranges of highest disease burden.

**Supplementary Information:**

The online version contains supplementary material available at 10.1186/s12916-021-02224-w.

## Background

Malaria is a vector-borne disease endemic to large parts of sub-Saharan Africa (SSA), where the predominant parasite that infects humans is *Plasmodium falciparum*. Exposure to bites from infected mosquitoes results in human infection which can be asymptomatic or develop into mild symptoms including fever, and a minority of infections progress to severe complications that might result in death [1, 2]. Immunity is dependent on the frequency of parasite exposure from birth and is thought to manifest at different periods in an individual’s lifetime for severe life-threatening disease, mild, self-limiting disease and asymptomatic blood-stage infection [3, 4]. The relationship between natural parasite exposure, immunity and the age-specific patterns of severe disease and death has been inadequately defined [1, 5, 6] but remains critical to understanding targeted intervention.

Reliable information on the age-specific patterns of malaria mortality are difficult to obtain in SSA [7] where most deaths occur in the community without medical or laboratory confirmation. Cause-of-death, therefore, depends entirely on responses from verbal autopsies (VA) administered to bereaved relatives [8]. Attribution of malaria as a cause of death using VA largely depends on reported fever during the terminal illness which lacks both sensitivity and specificity [9–11]. An alternative source of information is severe, life-threatening malaria which can be identified by clinical assessment [12–15]. Whilst not all severe malaria episodes reach the hospital, those that do provide important insights into the epidemiology of the life-threatening disease in the communities served by these hospitals.

Community-based parasite prevalence continues to be one important measure of malaria used in the epidemiological sub-national stratification for intervention decision-making [16, 17]. The *Plasmodium falciparum* parasite prevalence is measured in households or schools among asymptomatic individuals providing a quantity of the intensity of malaria transmission in a given locality and traditionally standardised to a single age group 2–10 years (*Pf*PR_2–10_) [17–20].

Twenty years ago, hospitalised malaria events were used to provide epidemiological descriptions of severe malaria across different malaria transmission ecologies in SSA [21–31] during an era of failing first-line treatment and widespread increases in parasite prevalence [17]. The relationship between community parasite prevalence and severe malaria outcomes continues to be based on hospital data generated over 20 years ago [32–35]. New data are needed to guide the appropriate age ranges for novel personal protection interventions linked to the expanded programme of immunisation (EPI). Intermittent presumptive treatment in infancy (IPTi) is currently targeted at children aged 2–11 months [36, 37] and RTS,S/R21 vaccination is targeted at children aged 5–24 months with protection estimated at around 36-month duration following a fourth dose [38–40]. Seasonal malaria chemoprevention (SMC) focuses predominantly on providing drugs to children below 5 years across seasonal transmission sites of the Sahel [41] and recently combined with RTS,S vaccination in this age group [42, 43].

The absence of contemporary empirical data on the age patterns of malaria mortality and severe disease across the range of transmission settings that characterise Africa today limits our ability to target novel interventions to maximise impact. Here we present a description of age and clinical epidemiology of malaria hospitalisation among children 1 month to 14 years of age presenting to 21 hospitals in East Africa that serve communities of varying parasite prevalence common to the sub-region since the introduction of artemisinin-based combination therapy and expanded vector control.

## Methods

### Study site selection

A secondary analysis was performed on data from hospitals that maintained continuous paediatric ward surveillance including malaria parasitology, purposively designed case record forms and documentation of patient residential addresses between 2006 and 2021. These were sentinel hospital sites for randomised controlled trials or sites for intensive clinical surveillance (Additional file 1: Fig. S1 & Additional file 2: Table S1) [44–66]. Twenty-one hospitals were included, which supported emergency care from 36 administrative areas and in over 49 separate hospital-site-time periods across East Africa (Additional file 1: Fig. S1 & Additional file 2: Table S1). Administrative areas were defined proximal to each hospital and represented as sub-counties in Kenya, councils in Tanzania and districts in Uganda. Address details of patients were reviewed to identify those from specific administrative areas. Site data covering more than one period were analysed as independent data items given interruptions in surveillance (Uganda) or reasoned temporal divisions in the data based on scaled introduction of free insecticide-treated nets (Kilifi and Rarieda, Kenya) or indoor-residual house-spraying (Tororo, Uganda), hereafter referred as site-time periods.

### Measures of transmission intensity

Parasite prevalence among residents of areas served by the 21 hospitals provided a categorical quantity of malaria exposure in each location. We have used temporally and spatially (sub-county, council or district) matched predicted estimates of *Plasmodium falciparum* parasite prevalence in children aged 2–10 years (*Pf*PR_2–10_), generated from Bayesian model-based spatiotemporal geostatistical analysis of > 18,000 empirical geocoded point surveys undertaken across East Africa since 2010 [64]. In brief, the Bayesian hierarchical space-time model was implemented through a stochastic partial differential equations approach using the Integrated Nested Laplace Approximations in R adjusting for climatic and ecological covariates priors to improve predictions of *Pf*PR_2–10_ at unsampled locations. Data were corrected to standardise surveys using rapid diagnostic tests (RDT) to microscopy using a Bayesian *probit* regression [67]. Computation used the full spatial and temporal range of the data and aimed at estimating the continuous posterior median of *Pf*PR_2–10_ at 1 × 1 km spatial resolutions for each year from 2010 and aggregated to the spatial extents of each administrative, hospital polygon and over the surveillance period of each hospital-catchment period. For sites pre-2010, similar, published geostatistical modelled approaches were used to obtain predicted *Pf*PR_2–10_ but these models did not include covariate priors nor correct RDT to microscopy in Kenya [65] and Tanzania [66]. We have elected to use categorical definitions of parasite prevalence used by national malaria programmes in East Africa to select interventions most likely to provide optimised, efficient use of malaria prevention strategies sub-nationally [65, 66, 68, 69]: low transmission intensity settings for *Pf*PR_2–10_ < 5%, low-moderate for *Pf*PR_2–10_ 5–9%, moderate for *Pf*PR_2–10_ 10–29%, and high for *Pf*PR_2–10_ ≥ 30%.

### Malaria case definitions

Children were included in the analysis if they were aged 1 month to 14 years, were resident in selected administrative areas and had evidence of laboratory-confirmed malaria infection on admission and a final discharge diagnosis of malaria following review of all available clinical and laboratory findings by hospital clinicians. At admission, information was documented for each patient on severe disease phenotypes including measured levels of consciousness, respiratory distress and anaemia (Additional file 2: Table S2) [15, 70, 71]. Cerebral malaria (CM) was defined at different sites using either the Blantyre Coma Score (BCS) < 3 (10 site-time periods) or documented neurological responsiveness based on the Alert, response to Voice, response to Pain, or Unconscious (AVPU) scale (39 site-time periods), where unconscious was regarded as equivalent to a BCS < 3. Severe malaria anaemia (SMA) was defined among children who had a haemoglobin (HB) < 5 g/dl. However, haemoglobin concentrations were not available among all admissions at all sites (Additional file 2: Table S2). Where HB was not available, information on whether clinicians ordered a blood transfusion was used as a secondary measure of SMA. SMA was defined based on HB among 11,671 admissions where a blood sample was taken. For a further 9,869 admissions where HB was not reported, blood transfusion ordered was indicative of SMA [72]. Respiratory distress (RD) was defined as observed deep breathing, a proxy for acidotic breathing, available at 43 sites. Data to assess other criteria for severe malaria (e.g. blood glucose or renal function) were not available and some children admitted for malaria may not have formally met the criteria for severe malaria. We included “all cause malaria admissions” as a group that would include both these groups as well as SMA, CM and RD. We included 21,540/46,076 (46.7%) of the malaria admissions from 43 site-time periods, where information on all three severe malaria phenotypes (SMA, RD and CM) were documented to demonstrate the overlap in these three phenotypes.

### Statistical analyses

A Bayesian generalised linear mixed model (GLMM) was developed to analyse how the age distribution of all-cause malaria admissions changed with parasite prevalence. Age was assumed to follow a Gamma distribution, with the average of this distribution modelled with a log-linear function of parasite prevalence. The random effect accounted for site-time deviations from the predicted mean. We compared a model with and without the log-linear effect of parasite prevalence to determine the statistical support for the inclusion of this term in the model using the widely applicable information criterion, WAIC [73]. Additionally, the age distribution of the three phenotypes (SMA, RD and CM) was assessed using a similar model form but included an additional random effect term to account for phenotype-specific site-time deviations. Models were fit in the Bayesian inference package Stan version 2.21.0 [74] interfaced through the statistical software R version 4.1.0 [75]. Six chains were run until all parameters achieved convergence (*R*≤1.01). Uncertainty was reported as 95% credible intervals (CIs). A detailed description of the model structure and selection procedure can be found in Additional file 3.

In the pilot implementation study in Ghana, Malawi and Kenya, the first dose of RTS,S vaccine is recommended in children aged 5–6 months, subsequent doses 2 and 3 given at respective infant EPI schedules and a 4th dose at 22–24 months [76]. IPTi is recommended to begin at 8–10 weeks with continued administration at repeated EPI attendances until the first birthday with at least 1 month between doses and up to 4 doses in the first year of life [36]. Additional studies have shown the efficacy of IPTi with protection age windows up to 15–24 months of life [77, 78]. To estimate the probability of all-cause malaria admission and the three phenotypes (SMA, RD and CM) occurring within the age groups 2–11 months and 2–23 months under different levels of transmission, the cumulative probability function of the Gamma distribution of the best fitting model was used. Protection from vaccines post-24 months and the possibilities of extending chemoprevention and vaccines up to 59 months of age were also considered.

## Results

**Fig. 1:**
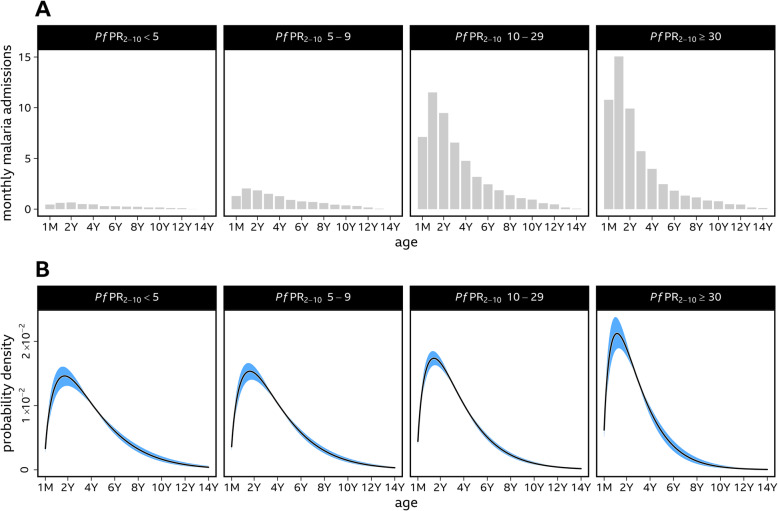
The age distribution of all cause malaria hospitalisation across discrete categoriesof community parasite prevalence.**Panel A**: The age-specific (binned into year groups) number of paediatric malaria admissions adjusted for the number of months of surveillance per transmission category. **Panel B**: The modelled age distribution of all-cause malaria admissions for each endemicity category is plotted with 95% credible intervals from a Gamma-distributed mixed effects model of malaria admissions

A total of 52,684 malaria admissions were documented at 21 hospitals covering 1,712 months of observation between 2006 and 2021. All children had a positive malaria slide, a primary discharge diagnosis of malaria, age documented and residential address identified. Forty-nine hospital site-time periods were spatially and temporally matched to the predicted *Pf*PR_2–10_ (Additional file 2: Table S1). The 49 site-time locations were characterised by a range of vector control approaches and coverage levels (Additional file 2: Table S1) and covered predicted malaria transmission conditions from < 1% *Pf*PR_2–10_ in Uganda and Kenya to the highest predicted transmission intensity described at Kisumu West 2015-18, Gem 2010-14, Alego-usonga 2010-14, Tororo 2012-15 and Apac 2017-18 where *Pf*PR_2–10_ exceeded 40% (Additional file 2: Table S1). No site-time locations covered contemporary transmission where predicted *Pf*PR_2–10_ was ≥50%. Twelve site-time data series were described as low transmission (< 5% *Pf*PR_2–10_), five were defined as low-moderate transmission (5–9% *Pf*PR_2–10_), 20 were moderate transmission (10–29% *Pf*PR_2–10_) and 12 were defined as high transmission (≥30% *Pf*PR_2–10_). When admissions were adjusted for the number of months of surveillance per transmission category, malaria hospital admission burdens were considerably lower in the low and low-to-moderate parasite transmission intensity settings compared to moderate and high transmission settings (Fig. 1A).

**Fig. 2:**
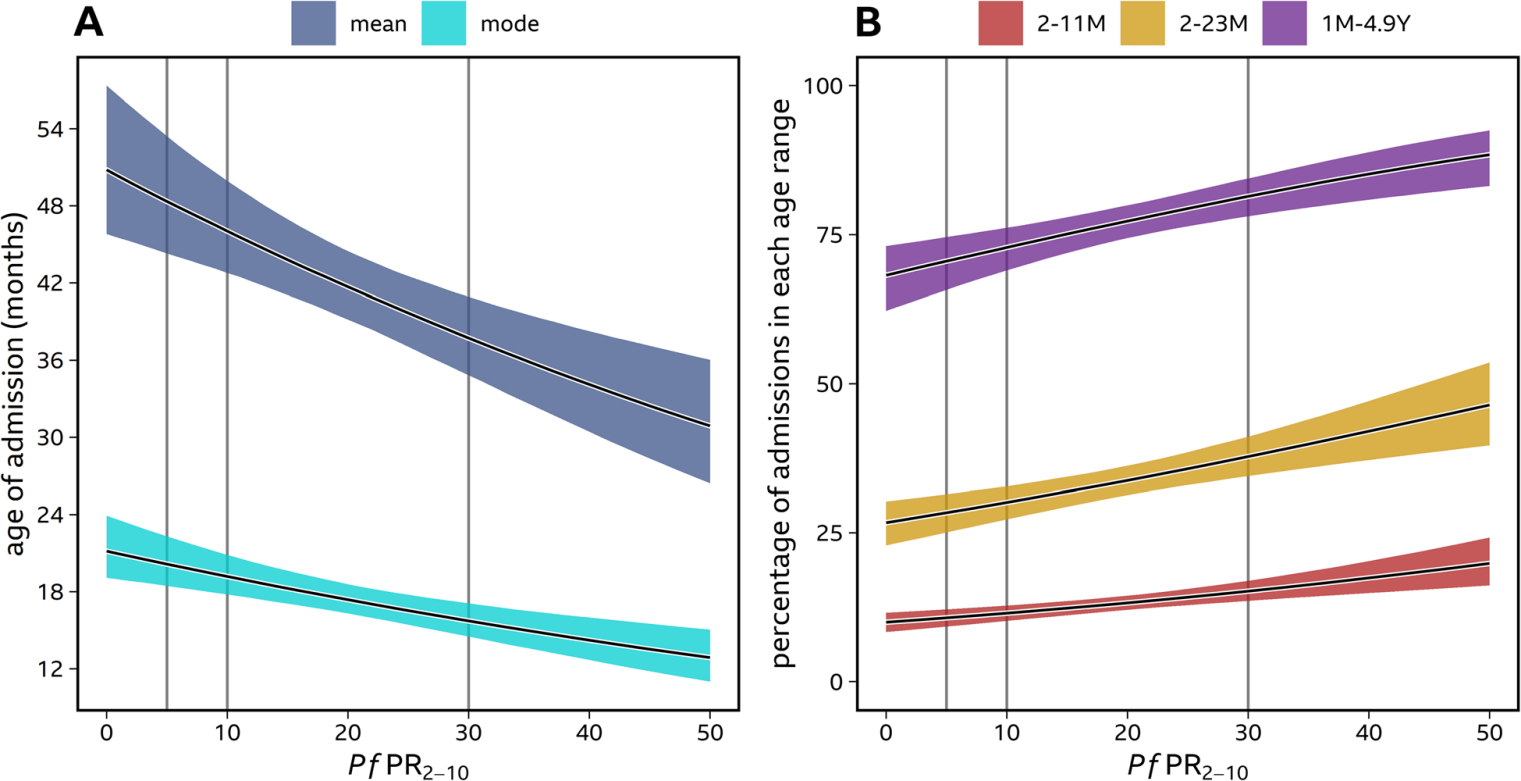
The modelled effect of parasite prevalence on the age characteristics of all-causemalaria admissions. **Panel A**, the change in the mean and most frequent age of admission is shown.**Panel B** gives effect of parasite prevalence on the probability of being admitted in one of three discrete age categories as calculated by integrating the Gamma density function across the age range

Including the effect of parasite prevalence in the age distribution model was supported by GLMM model selection (WAIC = 2.6) and the model fit for the individual site-time periods is shown in Additional file 1: Fig. S2. The modelled age distributions of all-cause malaria admission for each of the four discrete categories of *Pf*PR_2–10_ are shown in Fig. 1B. In addition, the modelled relationship between parasite prevalence and properties relating to the age distribution of malaria admissions are presented (Fig. 2). The average age of admission as predicted by the GLMM decreased as parasite prevalence increased (Figs. 1B and 2A), ranging from 49.5 months (95% *CI* 45.1, 55.4) at low transmission (< 5% *Pf*PR_2–10_) to 34.1 months (95% *CI* 30.4, 38.3) in the highest transmission classification (≥30% *Pf*PR_2–10_).

Across all transmission settings, admissions were concentrated in children under 5 years old (Figs. 1B and 2B), ranging from 69.4% (95% *CI* 64.0, 73.8) in the lowest transmission category (*Pf*PR_2–10_ < 5%) to 85.2% (95% *CI* 80.9, 88.9) in the highest transmission category (*Pf*PR_2–10_ ≥ 30%) as predicted from the cumulative probability function of the Gamma distribution. Furthermore, across all transmission settings, the probability of admitted children being aged 10 years and above was very low at 5.4% (95% *CI* 3.7, 7.9) in the lowest transmission category and 0.7% (95% *CI* 0.1, 1.6) in the highest transmission category (Fig. 1B). Under low transmission, 10.3% (95% *CI* 8.8, 11.8) of admitted children were predicted to be aged 2–11 months and 27.5% (95% *CI* 24.0, 30.8) to be children aged 2–23 months. In the low-moderate, the corresponding probabilities of admission were 11.1% (95% *CI* 9.7, 12.4) for 2–11 months and 29.2% (95% *CI* 26.2, 32.1) for 2–23 months. At levels of moderate transmission intensity, the probability of admitted children being aged 2–11 months was 13.2% (95% *CI* 12.1, 14.4) and 33.8% (95% *CI* 31.3, 36.2) for the age range 2–23 months. These values rose to 17.4% (95% *CI* 14.9, 20.2) and 42.0% (95% *CI* 37.2, 47.1), respectively, in the highest transmission intensity of *Pf*PR_2–10_ ≥ 30%.

**Fig. 3:**
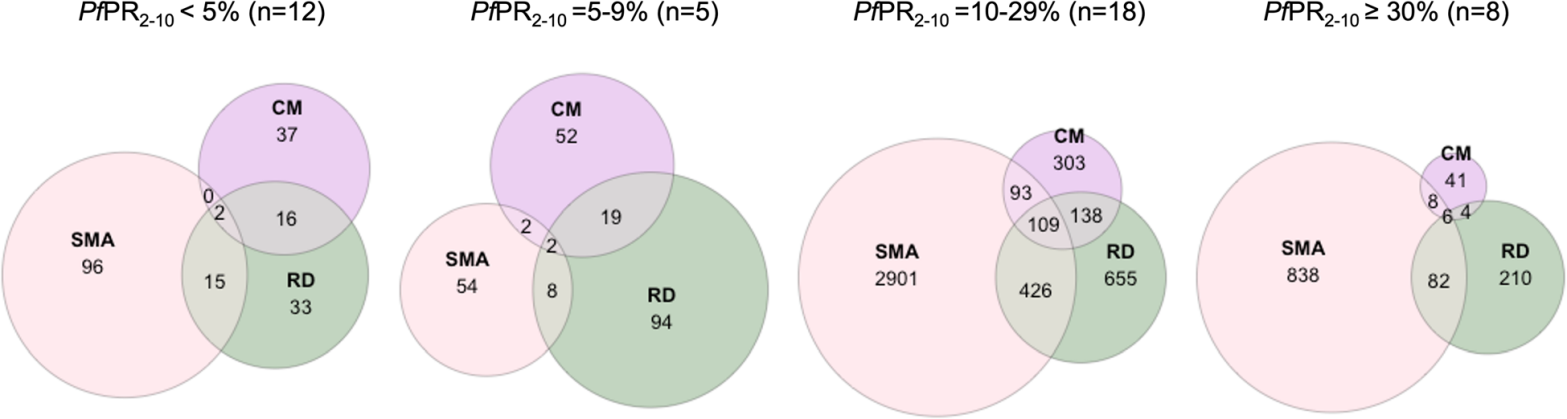
The overlap in severe malaria phenotypes across the four discrete categories of parasite prevalence. Severe malaria phenotypes were described among 21,541 admissions with complete clinical data from 43 site-time locations

Across the four discrete categories of *Pf*PR_2–10_, SMA was the most common phenotype, accounting for 74.3% (4642/6244) of all severe malaria admissions and in 76.3% and 78.6% in the moderate and high transmission settings, respectively (Fig. 3). RD was described in 1819 admissions and accounted for 29.1% of all severe malaria admissions across the combined transmission settings (Fig. 3). CM was described among 832 (13.3%) severe malaria admissions and was a much rarer event compared to SMA and RD; however, CM was relatively more common in the two lowest transmission categories albeit rare in absolute terms across all settings (Fig. 3).

The age patterns of the three severe malaria phenotypes showed similar trends in decreasing mean ages with increasing intensity of transmission classification, despite differences in the mean ages between phenotypes (Table 1). The mean age of SMA cases decreased from 44.6 months (95% *CI* 39.5, 50.6) in low transmission areas to 31.1 months (95% *CI* 27.4, 35.1) in areas of highest transmission (Table 1). In the low transmission areas, 74.5% (95% *CI* 68.4, 79.8) of SMA cases were reported below 5 years of age and 88.6% (95% *CI* 84.5, 92.0) in the high transmission areas (Table 1). RD followed a similar age pattern to SMA although children were overall younger in each transmission class, the mean age of RD cases decreased from 40.1 months (95% *CI* 35.2, 46.0) in the lowest transmission category to 27.9 months (95% *CI* 24.2, 32.4) in areas of high transmission (Table 1). The mean age in the lowest transmission category for CM was 56.2 months (95% *CI* 49.0, 64.9) and 39.1 months (95% *CI* 33.9, 45.1) in the highest transmission category. Across all severe phenotypes and all transmission classifications, the majority of admissions continued to occur among children under-5 years (Table 1). Children aged 2–23 months accounted for 30.7% (95% *CI* 26.2, 35.4) of all SMA cases in the low transmission category to 45.9% (95% *CI* 40.4, 51.7) in the highest transmission category. Similarly, RD had a higher proportion of cases in the 2–23-month age group in the high transmission setting (50.9%) compared to the low transmission setting (34.8%). CM was rarer among children aged 2–23 months in the low transmission setting (22.8%) compared to the highest transmission setting (35.8%) (Table 1).

**Table 1:** Characteristics of malaria admissions (1 month–14 years) by predicted *Pf*PR_2–10_ categories. Further details for each site-time period are provided in Additional file 2: Table S2

Indicators	***Pf***PR_**2–10**_ < 5%	***Pf***PR_**2–10**_ 5-9%	***Pf***PR_**2–10**_ 10–29%	***Pf***PR_**2–10**_ ≥ 30%	Overall
**No. of sites (date range)**	12 (2006–2021)	5 (2012–2021)	20 (2006–2021)	12 (2006–2021)	49 (2006–2021)
**Site-time in months**	447	158	736	371	1712
**All-cause malaria**	1431	1540	31885	17828	52684
**Mean age months (95%** ***CI*)**	49.5 (45.1, 55.4)	47.2 (43.5, 51.6)	41.7 (39.2, 44.5)	34.1 (30.4, 38.3)	42.1 (39.1, 45.2)
**Age-specific probabilities (%)**					
**2–11 age in months (95%** ***CI*)**	10.3 (8.8, 11.8)	11.1 (9.7, 12.4)	13.2 (12.1, 14.4)	17.4 (14.9, 20.2)	13.1 (11.8, 14.5)
**2–23 age in months (95%** ***CI*)**	27.5 (24.0, 30.8)	29.2 (26.2, 32.1)	33.8 (31.3, 36.2)	42.0 (37.2, 47.1)	33.4 (30.8, 36.3)
**Under 5 years (95%** ***CI*)**	69.4 (64.0, 73.8)	71.7 (67.4, 75.4)	77.3 (74.4, 79.9)	85.2 (80.9, 88.9)	76.9 (73.7, 79.9)
**Mortality (95%** ***CI*);¶** ***n*/*N*** (no. of sites)	3.2% (2.3, 4.3) 45/1406 (12)	2.0% (1.4, 2.9) 31/1518 (5)	1.8% (1.7, 2.0) 532/28774 (20)	1.0% (0.9, 1.2) 166/16672 (12)	1.5% (1.4, 1.7) 744/48370 (49)
Severe malaria anaemia* (%) ***n*/*N*** (no. of sites)	8.6% 115/1341 (12)	5.4% 66/1229 (5)	27.0% 3798/14088 (20)	14.8% 1281/8663 (12)	20.8% 5260/25321 (49)
**Mean age months (95%** ***CI*)**	44.6 (39.5, 50.6)	42.5 (38.1, 47.7)	37.7 (34.2, 41.5)	31.1 (27.4, 35.1)	39.0 (35.3, 42.7)
**Age-specific probabilities (%)**					
**2–11 age in months (95%** ***CI*)**	11.5 (9.4, 13.7)	12.3 (10.4, 14.5)	14.6 (12.7, 16.8)	19.1 (16.1, 22.6)	14.0 (12.2, 16.2)
**2–23 age in months (95%** ***CI*)**	30.7 (26.2, 35.4)	32.5 (28.2, 36.9)	37.4 (33.3, 41.6)	45.9 (40.4, 51.7)	36.0 (32.3, 40.2)
**Under 5 years (95%** ***CI*)**	74.5 (68.4, 79.8)	76.7 (71.3, 81.3)	81.8 (77.7, 85.5)	88.6 (84.5, 92.0)	80.3 (76.4, 84.2)
**Mortality (95%** ***CI*);¶** ***n*/*N*** (no. of sites)	8.3% (3.9, 15.2) 9/108 (12)	9.5% (3.6, 19.6) 6/63 (5)	5.3% (4.5, 6.1) 170/3209 (20)	4.2% (3.1, 5.5) 49/1169 (12)	5.1% (4.5, 5.8) 234/4549 (49)
**Respiratory distress (%)** ***n*/*N*** (no. of sites)	6.0% 85/1426 (12)	10.6% 162/1531 (5)	9.5% 2740/28808 (18)	4.8% 615/12884 (8)	8.1% 3602/44649 (43)
**Mean age months (95%** ***CI*)**	40.1 (35.2, 46.0)	38.1 (33.9, 43.4)	33.8 (30.3, 38.2)	27.9 (24.2, 32.4)	33.7 (30.3, 37.1)
**Age-specific probabilities (%)**					
**2–11 age in months (95%** ***CI*)**	13.4 (10.9, 16.1)	14.4 (11.9, 17.0)	17.0 (14.3, 19.8)	22.1 (18.1, 26.4)	17.2 (15.0, 19.8)
**2–23 age in months (95%** ***CI*)**	34.8 (29.5, 40.3)	36.8 (31.6, 42.0)	42.0 (36.8, 47.0)	50.9 (44.0, 57.7)	42.2 (38.0, 47.0)
**Under 5 years (95%** ***CI*)**	79.2 (73.0, 84.4)	81.2 (75.7, 85.8)	85.8 (81.2, 89.3)	91.6 (87.2, 94.6)	85.9 (82.3, 89.3)
**Mortality (95%** ***CI*);¶** ***n*/*N*** (no. of sites)	12.9% (6.6, 22.0) 11/85 (11)	6.3% (3.1, 11.3) 10/158 (3)	6.1% (5.1, 7.1) 147/2427 (17)	5.8% (4.0, 8.1) 32/553 (8)	6.2% (5.4, 7.1) 200/3223 (39)
**Cerebral malaria (%)** ***n*/*N*** (no. of sites)	4.2% 60/1423 (12)	5.3% 81/1530 (5)	3.1% 945/30494 (20)	0.6% 105/17052 (12)	2.4% 1191/50499 (49)
**Mean age months (95%** ***CI*)**	56.2 (49.0, 64.9)	53.5 (47.1, 61.1)	47.4 (42.1, 53.5)	39.1 (33.9, 45.1)	48.7 (43.3, 54.1)
**Age-specific probabilities (%)**					
**2–11 age in months (95% CI)**	8.1 (6.4, 10.0)	8.7 (7.0, 10.6)	10.5 (8.7, 12.5)	13.9 (11.2, 17.1)	10.1 (8.6, 12.1)
**2–23 age in months (95% CI)**	22.8 (18.7, 27.3)	24.4 (20.3, 28.6)	28.4 (24.3, 32.9)	35.8 (30.1, 42.0)	27.6 (24.0, 31.9)
**Under 5 years (95% CI)**	63.2 (55.9, 70.0)	65.6 (58.9, 71.9)	71.5 (65.6, 77.0)	80.3 (73.9, 85.8)	70.3 (65.0, 75.7)
**Mortality (95%** ***CI*);¶** ***n*/*N*** (no. of sites)	13.3% (5.9, 24.6) 8/60 (9)	13.8% (7.1, 23.3) 11/80 (4)	13.7% (11.4, 16.2) 115/840 (17)	14.4% (7.9, 23.4) 13/90 (11)	13.7% (11.7, 15.9) 147/1070 (41)

*Severe malaria anaemia was defined as a composite of HB < 5 g/dl and blood transfusion ordered

**¶**A chi-square test was used to test the equality of case fatality across the transmission categories and the *p*-values are reported in the main text

Among the 52,684 malaria hospitalisations, discharge outcomes were not documented in 2611 (5.0%) individuals where guardians discharged patients against medical advice, 272 (0.5%) were transferred to another facility and 1431 (2.7%) where no details were documented. Among 48370 patients where the discharge outcome was documented, 744 (1.6%) children died. There were differences in case fatalities between transmission settings (*p* < 0.001), ranging from 3.2% in the lowest transmission category where overall disease burdens were low to 1.0% in the highest transmission, high burden category (Table 1). In the pooled dataset across all transmission settings, the case fatality for each of the three severe malaria phenotypes was different: SMA (5.1%: 95% *CI* 4.5, 5.8), RD (6.2%: 95% *CI* 5.4, 7.1) and CM (13.7%: 95% *CI* 11.7, 15.9) (Table 1). Although SMA mortality was highest in the two lowest transmission categories (8.3–9.5%), the case fatality of SMA did not significantly vary across transmission classifications (*p* = 0.070). Similarly, the case fatality for RD was highest in the lowest transmission category (12.9%) although there were no differences across the four categories of *Pf*PR_2–10_ (*p* = 0.076). Case fatality for CM was high (13.3–14.4%) and similar across all the endemicity classes (*p* = 0.997) (Table 1).

## Discussion

We have analysed the age patterns of malaria hospitalisation in over 52,000 admissions to emergency care wards at 21 hospitals over 49 different site-time-specific periods in East Africa linked to predicted community-based malaria prevalence. This series represents one of the largest, contemporary descriptions of the epidemiology of severe, hospitalised malaria in Africa. Consistent with earlier descriptions during the 1990s and early 2000s [28–30], the numbers of children admitted with a parasitologically confirmed, primary diagnosis of malaria declines rapidly throughout the first 10 years of life and remains uncommon thereafter (Fig. 1A, B). This age-specific decline in admissions with malaria was broadly similar across all four discrete classes of community prevalence, used to develop sub-national epidemiological stratifications in East Africa. Children admitted with malaria tended to be younger in higher transmission intensity classifications (≥10% *Pf*PR_2–10_) compared to those admitted from areas with lower transmission intensity (< 10% *Pf*PR_2–10_) (Table 1). Most admissions (69–85%) across all transmission settings were among children below 5 years of age supporting the current emphasis across SSA of targeting young children for personal protection measures. However, it is important to recognise that when data were corrected for the sampling time-effort to highlight the monthly hospital burdens across transmission sites, malaria admissions are much lower on average in areas of *Pf*PR_2–10_ 5–9% compared to transmission settings where predicted *Pf*PR_2–10_ is ≥10%, and even less common in areas where *Pf*PR_2–10_ is < 5% (Fig. 1A). These findings among a wider range of hospitals, transmission settings, and age inclusion are consistent with our recently published observations based on 6506 malaria hospitalisations among children aged 3 months to 9 years which were restricted to communities immediately proximal to sentinel hospitals [72]. Both data series and analyses highlight that the highest hospital disease burden warranting concerted, high coverage of personal infection prevention strategies remain in areas where *Pf*PR_2–10_ is ≥10%, and hospital disease burdens below this threshold may be less cost-effective for sub-national targeting of additional individual-level disease prevention strategies in Africa.

Two individual-level protection strategies that have the potential to augment the current emphasis on vector control in high transmission areas (*Pf*PR_2-10_ ≥ 10%) of East Africa are IPTi and RTS,S/R21 vaccination. Both interventions are aimed at operationally capitalising on successful existing EPI programme strategies (IPTi) or adapted EPI (malaria vaccines). Here we show that malaria hospitalisation among all admissions below 15 years occurring between 2 and 23 months was 34% in areas where predicted *Pf*PR_2–10_ was 10–29% rising to 42% where predicted *Pf*PR_2–10_ ≥ 30%. The probability of an admitted child being aged 2–11 months was 13% rising to 17% when predicted *Pf*PR_2–10_ was 10–29% and ≥ 30%, respectively, in support of the approach of targeting young children aged 2–23 months in transmission settings where *Pf*PR_2–10_ is ≥10%. In 2019, there were 125/366 (34%) decentralised administrative areas in East Africa that would benefit from additional personal protection offered through expanded EPI services, where predicted *Pf*PR_2–10_ was ≥10%, covering 2.3 million children aged less than 2 years [64].

Malaria admission above the age of 10 years was relatively uncommon at all transmission sites. There are several, not mutually exclusive, potential explanations as to why malaria hospitalisation becomes uncommon in late childhood across a wide range of transmission intensities. It may be that few parasite exposures result in the development of functional immune response to subsequent malaria hospitalisation [79–81]. Independent of exposure, age per se may confer protection against malaria hospitalisation/severe disease [82] and symptoms may be more easily recognisable among older children able to report symptoms rapidly, increasing opportunities for accessing effective treatment promptly. Whatever the underlying mechanism, our data indicate the rapid decline in severe disease in late childhood and early adolescence. This observation is inconsistent with the modelled global burden of disease estimates of malaria mortality that suggest that 22–58% of all deaths in SSA occur after the 15th birthday because of poor sensitivity and specificity of VA which these models heavily rely on [83–85].

We view malaria hospitalisation as a severe morbid event, distinct from ambulatory out-patient treatable malaria. Being admitted to a hospital suggests physicians are concerned about outcomes, and an admission leads to collateral consequences for patients’ families and the health system. However, a strict definition of severe, life-threatening malaria encompasses multiple specific pathologies defined for the use of clinical management [15] and outcome analysis during randomised trials [71]. In our series, SMA was the most common of the three severe malaria phenotypes, accounting for 74% of all severe admissions across all sites and the most frequent syndrome in each of the four transmission intensity classifications (Fig. 3). CM is a much rarer severe disease phenotype, although relatively more important in the lowest transmission settings (*Pf*PR_2–10_ < 5%) (Table 1; Fig. 3). CM tends to present among older children compared to SMA and RD across all transmission settings (Table 1) and the mean age of CM, SMA and RD increases significantly from the highest transmission setting to the lowest, consistent with all-cause malaria hospitalisation (Table 1). We were unable to assess other WHO criteria for severe malaria.

Mortality among malaria hospitalisation in East Africa ranged from 1% in the high transmission, high admission burden settings, to 3.2% in the lowest transmission, low admission burden settings (Table 1). Not surprising, the case fatalities were much higher in the severe disease phenotypes: 5.1% and 6.3% among the common phenotypes of SMA and RD, respectively, and 13.7% among the rarer phenotype of CM (Table 1). SMA and RD case fatalities were higher as transmission intensity declined whilst CM case fatality rates were similar, and high, across all transmission classifications (Table 1). Areas that experience low transmission and consequent lower disease burdens may be less well prepared for emergency treatment of severe malaria, and these areas also tend to have more CM than SMA and RD. In-hospital mortality from severe malaria remains high in East Africa and whilst hospital mortality may provide some epidemiological insights into cause-specific mortality burdens in the community [86], they also depend on the quality and timeliness of in-patient emergency care and delay in patient presentation [13].

Unlike our work defining hospital admission rates that purposively chose rural communities within easy reach of the hospital and no competing local admission facilities [72], the present analysis included a much wider community where distance to the hospital and use of other hospitals might have been different between hospital recruitment sites. The influence of travel time, hospital choice, pre-treatment triage at lower-level facilities and the age of hospital admission for malaria remains poorly defined [87]. Here we have presumed equal accessibility for all children aged less than 15 years within each transmission defined location, between sites and with time. To examine the possible influence of access delay, we analysed the duration of fever prior to admission by age and transmission setting, where overall 53% of all malaria presentations occurred with 48 h of reported fever onset and no significant differences between age groups or transmission settings (Additional file 1: Fig. S3).

Sites included in this analysis were those with stable perennial transmission in East Africa, as such we did not include traditionally acutely seasonal transmission settings more common in semi-arid areas of West Africa [29, 30, 88]. The influence of seasonal, pulsed parasite exposure on the age-specific patterns of hospitalised malaria might be very different and merits wider investigation in these areas of Africa.

We consider properly resourced, staff training, laboratory support and electronic data capture tools as essential to defining the clinical epidemiology of severe malaria and may serve as barometers of community-level transmission intensity to target interventions and ultimately used to monitor impact [28, 38, 86, 89]. We have shown that hospital burdens and age at admission scale with increasing levels of community-based parasite transmission intensity. We have defaulted to using classifications of community prevalence used by national programmes to provide information on targeting intervention combinations for prevention. With adequate resources, hospital surveillance alone could be used to decide on maximising age targets for intervention, circumventing the need for complex modelled predictions of community parasite prevalence. Decisions on the implementation of new malaria strategies must be tailored to the local health systems and epidemiological context. Investing in sentinel hospital surveillance would be an important layer of data to consider in all future sub-national, intervention stratification.

## Conclusion

Targeting chemoprevention or vaccination programmes to areas where community-based parasite prevalence is ≥10% is likely to cover the highest hospital burden. Extending chemoprevention e.g intermittent presumptive treatment in infancy to children aged 2–23 months and current vaccine age eligibility would target the most vulnerable age groups for malaria hospitalisation and the dominant severe disease phenotype of SMA.

## Supplementary Information

**Additional file 1.** Supplementary figures.

**Additional file 2.** Supplementary tables.

**Additional file 3.** Supplementary methods.

## Data Availability

Data used in this analysis have been curated and uploaded to the Harvard Dataverse [90]. Correspondence and requests for materials should be addressed to the KEMRI Wellcome Data Governance Committee (dgc@kemri-wellcome.org). These data are available through a formal requesting process to the KEMRI Institutional Data Access/Ethics Committee. Guideline details can be found on the KEMRI Wellcome website (https://kemri-wellcome.org/aboutus/#ChildVerticalTab_15).

## Abbreviations

AVPU: Alert, response to voice, response to pain, or unconscious
BCS: Blantyre Coma Score
CM: Cerebral malaria
EPI: Expanded programme of immunisation
GLMM: Generalised linear mixed model
HB: Haemoglobin
IPTi: Intermittent presumptive treatment in infancy
*Pf*PR_2–10_: *Plasmodium falciparum* parasite prevalence in children aged 2–10 years
RD: Respiratory distress
RDT: Rapid diagnostic tests
SMA: Severe malaria anaemia
SMC: Seasonal malaria chemoprevention
SSA: Sub-Saharan Africa
VA: Verbal autopsies
WAIC: Widely applicable information criterion
