# Supplementary material for: Malaria hospitalisation in East Africa: age, phenotype and transmission intensity
Source: BMC Med. 2022 Jan 27;20:28. doi: 10.1186/s12916-021-02224-w (PMC8793189; doi:10.1186/s12916-021-02224-w)
Supplement: Supplementary file 1 — Additional file 1. Supplementary figures. [file 12916_2021_2224_MOESM1_ESM.docx]

**Malaria hospitalisation in East Africa: age, phenotype and transmission intensity**

Alice Kamau, Robert S Paton, Samuel Akech, Arthur Mpimbaza, Cynthia Khazenzi, Morris Ogweru, Eda Mumo, Victor A Alegana, Ambrose Agweyu, Neema Mturi, Shebe Mohammed, Godfrey Bigogo, Allan Audi, James Kapisi, Asadu Sserwanga, Jane F Namuganga, Simon Kariuki, Nancy A Otieno, Bryan O Nyawanda, Ally Olotu, Athuman Thabit, Nayha Salim, Salim Abdulla, Amina F Mohamed, George Mtove, Hugh Reyburn, Sunetra Gupta, José Lourenço, Philip Bejon, Robert W Snow

**Additional file 1: Supplementary figures**

**
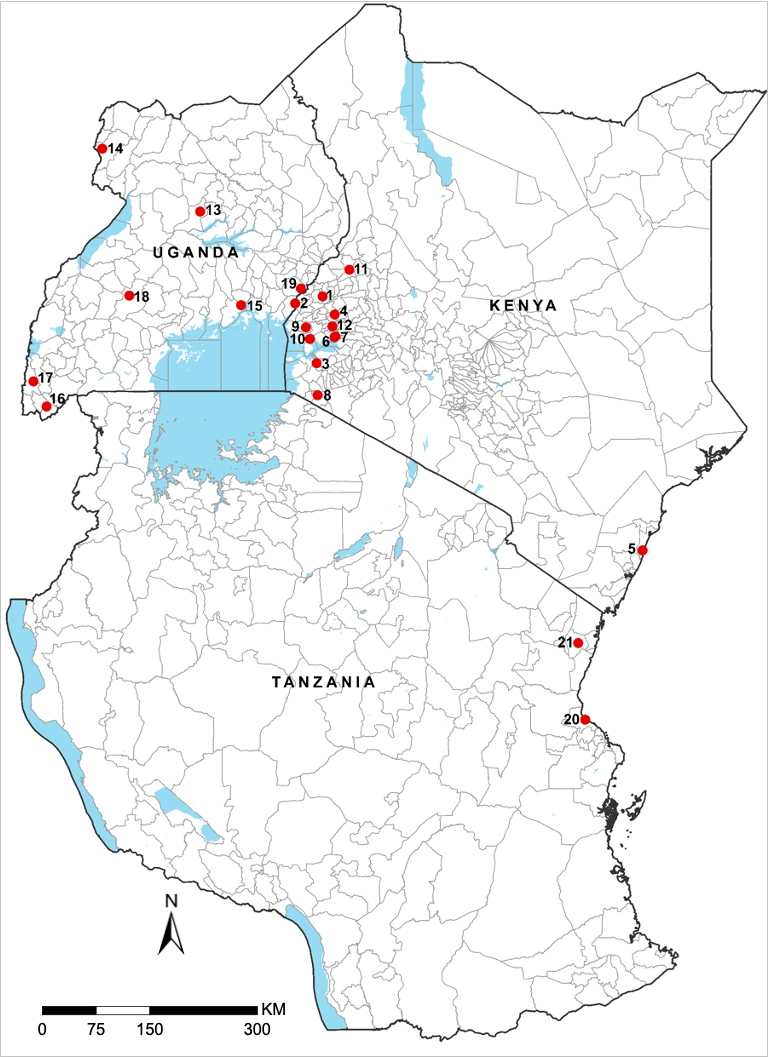
**

**Fig. S1: Geographical location of the 21 surveillance hospitals (red dots) in Kenya, Uganda and Tanzania.** The grey bounded polygons represent sub-counties in Kenya, districts in Uganda and councils in Tanzania. The numbering represents: Bungoma County Referral Hospital (1), Busia County Referral Hospital (2), Homa Bay County Referral Hospital (3), Kakamega County Teaching and Referral Hospital (4), Kilifi County Hospital (5), Kisumu County Hospital (6), Jaramogi Oginga Odinga Teaching and Referral Hospital (7), Migori County Referral Hospital (8), Siaya County Referral Hospital (9), St Elizabeth Lwak Mission Hospital (10), Kitale County Referral Hospital (11), Vihiga County Referral Hospital (12), Apac General Hospital (13), Arua Regional Referral Hospital (14), Jinja Referral Hospital (15), Kabale Regional Referral Hospital (16), Kambuga General Hospital (17), Mubende Referral Hospital (18), Tororo General Hospital (19), Bagamoyo District Hospital (20), Tuele Hospital (21).

**
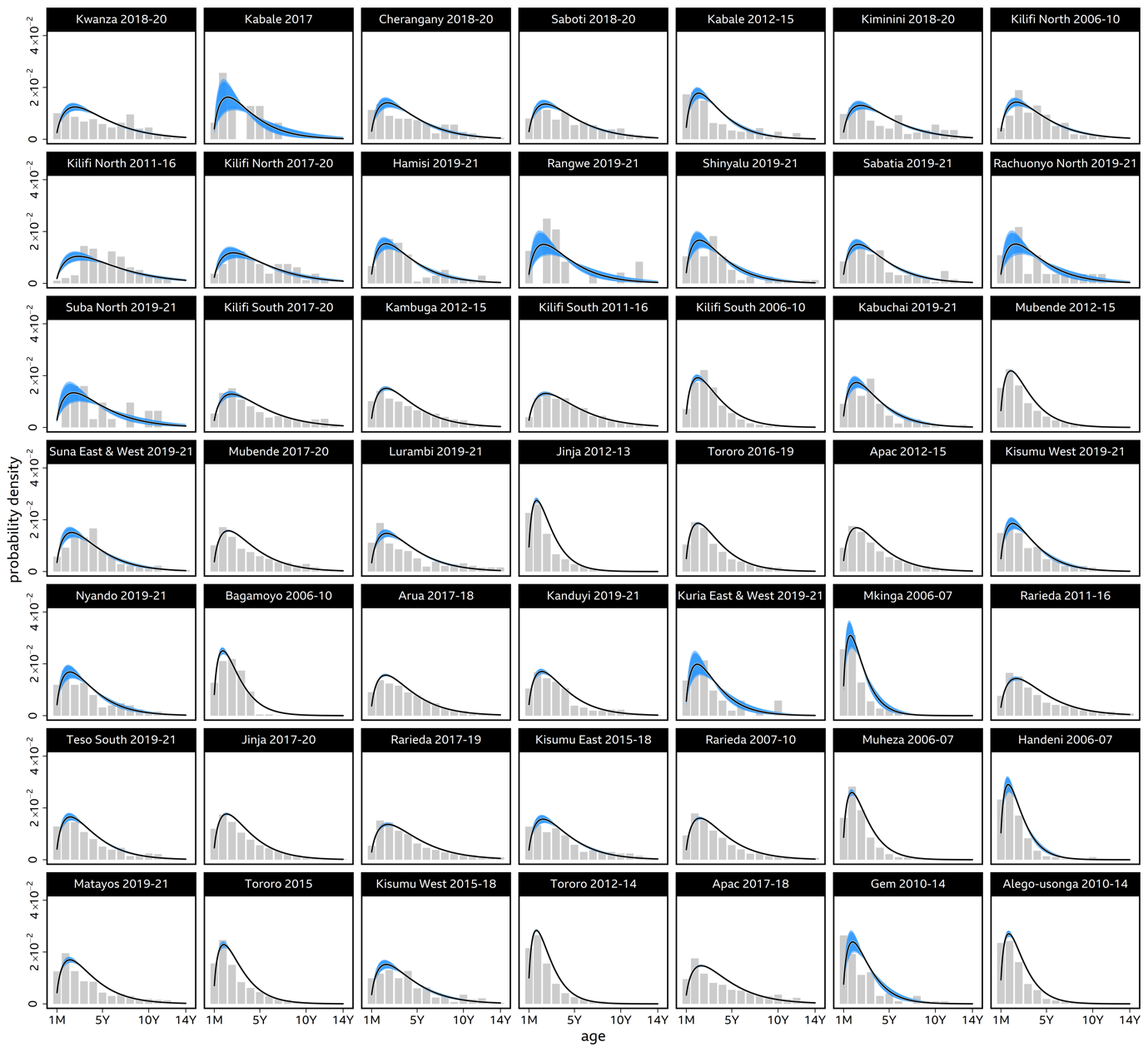
**

**Fig. S2: Site specific age distribution of all cause malaria hospitalisation (observed and predicted), ordered from lowest to highest predicted parasite prevalence.** Blue lines and intervals give the model fit for each site-time period, including the random effect of hospital site-time period correction on the age distribution. The grey bars give the observed distribution of all cause malaria hospitalisation.


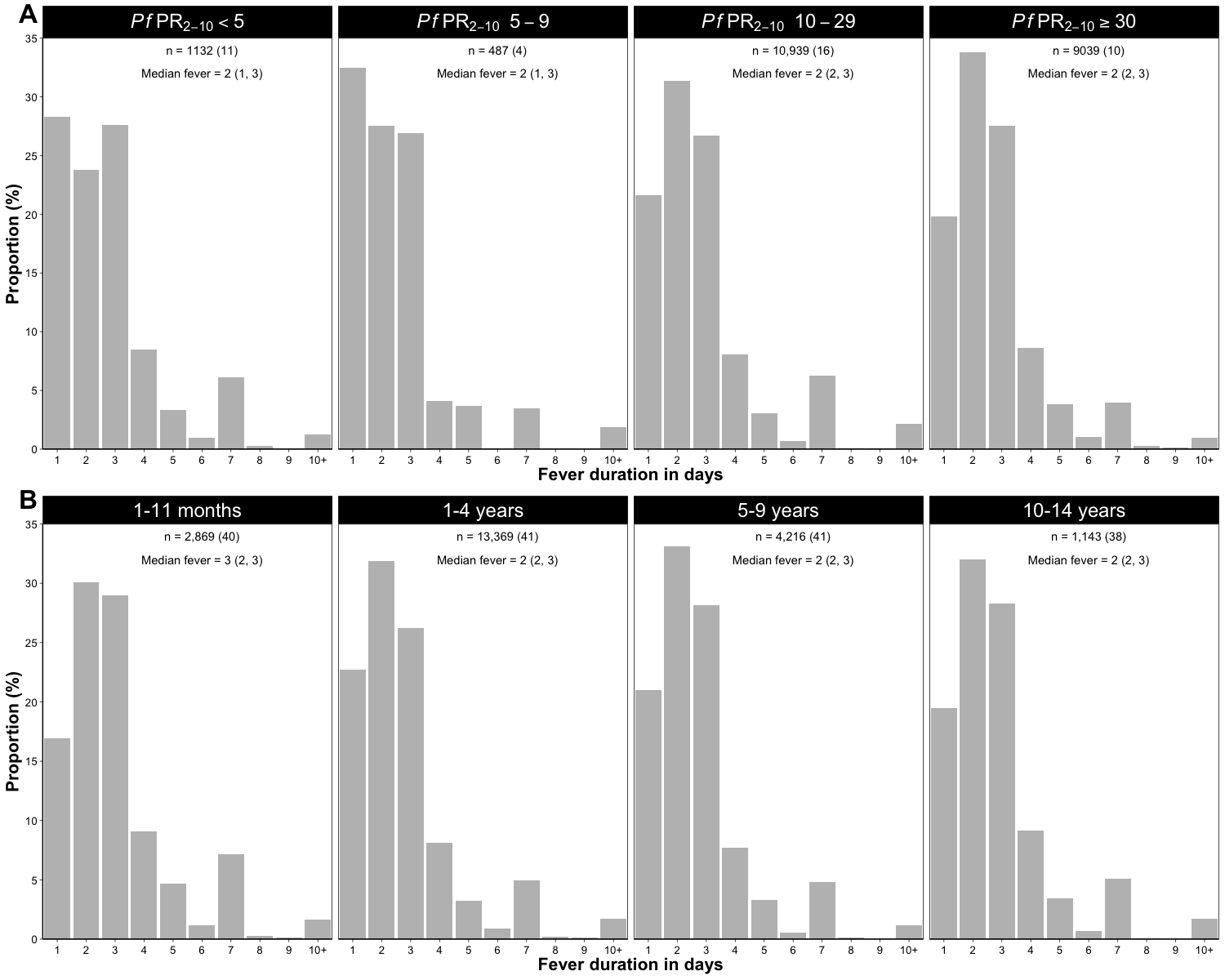


**Fig. S3: Fever duration for all cause malaria hospitalisation. Panel A:** The duration of fever prior to admission per transmission category. Reported n is the number of observations with fever duration and the number is brackets is the number of site-time periods included. The median fever duration and the IQR are also reported for each transmission category. There were no differences in fever duration across all transmission categories (p= 0.8273). **Panel B:** Fever duration prior to admission in four age groups; n, the number is brackets and median fever duration represents the same information reported in Panel A. There was little variability in the median fever duration across the four age groups (p=0.0875)
