# Supplementary material for: Malaria hospitalisation in East Africa: age, phenotype and transmission intensity
Source: BMC Med. 2022 Jan 27;20:28. doi: 10.1186/s12916-021-02224-w (PMC8793189; doi:10.1186/s12916-021-02224-w)
Supplement: Supplementary file 2 — Additional file 2. Supplementary tables. [file 12916_2021_2224_MOESM2_ESM.docx]

**Malaria hospitalisation in East Africa: age, phenotype and transmission intensity**

Alice Kamau, Robert S Paton, Samuel Akech, Arthur Mpimbaza, Cynthia Khazenzi, Morris Ogweru, Eda Mumo, Victor A Alegana, Ambrose Agweyu, Neema Mturi, Shebe Mohammed, Godfrey Bigogo, Allan Audi, James Kapisi, Asadu Sserwanga, Jane F Namuganga, Simon Kariuki, Nancy A Otieno, Bryan O Nyawanda, Ally Olotu, Athuman Thabit, Nayha Salim, Salim Abdulla, Amina F Mohamed, George Mtove, Hugh Reyburn, Sunetra Gupta, José Lourenço, Philip Bejon, Robert W Snow

**Additional file 2: Supplementary tables**

**Table S1: Site specific descriptions**

| **Site, dates (months of surveillance)** | **Map code** | **Insecticide net coverage in children** | **Indoor Residual House spraying dates** | ***Pf*PR_2-10_ ^d^** | **Main surveillance citation** |
| --- | --- | --- | --- | --- | --- |
| Kabuchai, Kenya |  |  |  |  |  |
| 2019-21 (24) | 1 | 57% ^a^ | None | 11.4% | 44 |
| Kanduyi, Kenya |  |  |  |  |  |
| 2019-21 (24) | 1 | 57% ^a^ | None | 25.2% | 44 |
| Matayos, Kenya |  |  |  |  |  |
| 2019-21 (28) | 2 | 62% ^a^ | None | 37.3% | 45-47 |
| Teso South, Kenya |  |  |  |  |  |
| 2019-21 (28) | 2 | 62% ^a^ | None | 28.6% | 45-47 |
| Rachuonyo North, Kenya |  |  |  |  |  |
| 2019-21 (24) | 3 | 76% ^a^ | Annually (2018-2021) | 6.5% | 44 |
| Rangwe, Kenya |  |  |  |  |  |
| 2019-21 (24) | 3 | 76% ^a^ | Annually (2018-2021) | 3.6% | 44 |
| Suba North, Kenya |  |  |  |  |  |
| 2019-21 (24) | 3 | 76% ^a^ | Annually (2018-2021) | 6.7% | 44 |
| Lurambi, Kenya |  |  |  |  |  |
| 2019-21 (28) | 4 | 59% ^a^ | None | 14.5% | 45-47 |
| Shinyalu, Kenya |  |  |  |  |  |
| 2019-21 (28) | 4 | 59% ^a^ | None | 3.8% | 45-47 |
| Kilifi North, Kenya |  |  |  |  |  |
| 2006-10 (60) | 5 | 33% ^b^ | None | 1.6% | 48-50 |
| 2011-16 (71) |  | 48% ^b^ | None | 1.7% |  |
| 2017-20 (36) |  | 45% ^b^ | None | 2.2% |  |
| Kilifi South, Kenya |  |  |  |  |  |
| 2006-10 (60) | 5 | 74% ^b^ | None | 10.8% | 48-50 |
| 2011-16 (71) |  | 66% ^b^ | None | 10.4% |  |
| 2017-20 (36) |  | 78% ^b^ | None | 7.3% |  |
| Kisumu East, Kenya |  |  |  |  |  |
| 2015-18 (25) | 6 | 73% ^a^ | None | 31.9% | 45-47 |
| Kisumu West, Kenya |  |  |  |  |  |
| 2015-18 (25) | 6 | 73% ^a^ | None | 42.2% | 45-47 |
| Kisumu West, Kenya |  |  |  |  |  |
| 2019-21 (23) | 7 | 64% ^a^ | None | 18.4% | 44 |
| Kisumu Nyando, Kenya |  |  |  |  |  |
| 2019-21 (28) | 7 | 81% ^a^ | None | 18.4% | 44 |
| Kuria East & West, Kenya |  |  |  |  |  |
| 2019-21 (19) | 8 | 71% ^a^ | None | 25.4% | 47 |
| Suna East & West, Kenya |  |  |  |  |  |
| 2019-21 (19) | 8 | 71% ^a^ | Annually (2017-2021) | 14.1% | 47 |
| Alego-usonga, Kenya |  |  |  |  |  |
| 2010-14 (60) | 9 | 55% ^c^ | None | 48.7% | 51, 52 |
| Gem, Kenya |  |  |  |  |  |
| 2010-14 (60) | 9 | 55% ^c^ | None | 45.9% | 51, 52 |
| Rarieda, Kenya |  |  |  |  | 53, 54 |
| 2007-10 (48) | 10 | 64% ^a^ | None | 31.9% |  |
| 2011-16 (72) |  |  | None | 26.4% |  |
| 2017-19 (32) |  |  | None | 30.7% |  |
| Cherangany, Kenya |  |  |  |  |  |
| 2018-20 (35) | 11 | NA | None | 0.4% | 45-47 |
| Kiminini, Kenya |  |  |  |  |  |
| 2018-20 (35) | 11 | NA | None | 0.8% | 45-47 |
| Kwanza, Kenya |  |  |  |  |  |
| 2018-20 (35) | 11 | NA | None | 0.3% | 45-47 |
| Saboti, Kenya |  |  |  |  |  |
| 2018-20 (35) | 11 | NA | None | 0.5% | 45-47 |

**Table S1 continued**

| **Site, dates (months of surveillance)** | **Map code** | **Insecticide net coverage in children** | **Indoor Residual House spraying dates** | ***Pf*PR_2-10_ ^d^** | **Main surveillance citation** |
| --- | --- | --- | --- | --- | --- |
| Hamisi, Kenya |  |  |  |  |  |
| 2019-21 (28) | 12 | 55% ^a^ | None | 3.6% | 45-47 |
| Sabatia, Kenya |  |  |  |  |  |
| 2019-21 (28) | 12 | 55% ^a^ | None | 6.1% | 45-47 |
| Apac, Uganda |  |  |  |  |  |
| 2012-15 (48) | 13 | 54% ^c^ | Annually  (2010- 2014) | 16.8% | 55-57 |
| 2017-18 (21) |  | 24% ^a^ | 2017 | 45.7% |  |
| Arua, Uganda |  |  |  |  |  |
| 2017-18 (24) | 14 | 67% ^c^ | None | 23.8% | 57 |
| Jinja, Uganda |  |  |  |  |  |
| 2012-13 (23) | 15 | 32% ^b^ | None | 15.1% | 55-57 |
| 2017-20 (40) |  | 54% ^a^ | None | 29.6% |  |
| Kabale, Uganda |  |  |  |  |  |
| 2012-15 (48) | 16 | 55% ^c^ | 2006, 2007 | 0.8% | 55-57 |
| 2017 (12) |  | 89% ^a^ | 2006, 2007 | 0.4% |  |
| Kambuga, Uganda |  |  |  |  |  |
| 2012-15 (46) | 17 | 63% ^c^ | None | 8.2% | 55 |
| Mubende, Uganda |  |  |  |  |  |
| 2012-15 (48) | 18 | 50% ^c^ | None | 13.3% | 55-57 |
| 2017-20 (40) |  | 42% ^a^ | None | 14.2% |  |
| Tororo, Uganda |  |  |  |  |  |
| 2012-14 (36) | 19 | 58% ^b^ | None | 44.1% | 55-58 |
| 2015 (12) |  | 58% ^b^ | 3 rounds in 2015 | 42.1% |  |
| 2016-19 (48) |  | 47% ^a^ | Annually  (2016-2019) | 16.2% |  |
| Bagamoyo, Tanzania |  |  |  |  |  |
| 2006-10 (57) | 20 | 40% ^c^ | None | 22.5% | 59-61 |
| Handeni, Tanzania |  |  |  |  |  |
| 2006-07 (12) | 21 | 27% ^c^ | None | 34.4% | 62, 63 |
| Mkinga, Tanzania |  |  |  |  |  |
| 2006-07 (12) | 21 | 27% ^c^ | None | 25.7% | 62, 63 |
| Muheza, Tanzania |  |  |  |  |  |
| 2006-07 (12) | 21 | 27% ^c^ | None | 33.5% | 62, 63 |

a. Long-lasting insecticide treated net (LLIN) use defined during school surveys undertaken within the catchment area during the surveillance period

b. LLIN use determined from community-based household surveys within the catchment areas during the surveillance period

c. LLIN use defined for the wider district that the catchment area was located in during national demographic and health surveys, or malaria indicator household surveys undertaken close to the surveillance period.

d. In 42 site-time periods, *Pf*PR_2-10_ predictions were obtained from Alegana et al. (2021) [64]. In Kilifi North 2006-10, Kilifi South 2006-10 and Rarieda 2007-10, *Pf*PR_2-10_ predictions were obtained from Macharia et al. (2018) [65] and in Bagamoyo 2006-10, Handeni 2006-07, Mkinga 2006-07 and Muheza 2006-07 *Pf*PR_2-10_ predictions were obtained from Runge et al. (2020) [66]. In 3 sites, Kilifi North 2011-16, Kilifi South 2011-16 and Rarieda 2011-16, where the predicted *Pf*PR_2-10_ overlapped, predictions by Alegana et al. (2021) [64] were 1.9%, 10.4% and 26.4%, respectively, compared to predictions by Macharia et al. (2018) [65] which were 1.8%, 9.6% and 26.5%, respectively. Despite differences in the model form, this provides some assurance in the congruence between the methods.

**Table S2: Site specific clinical indicators.**

| **Site, dates** | **No. malaria admissions (definition)** | **Hb**  **(coverage %)** | **Reported transfusion ordered (coverage %)** | **SMA†**  **(coverage %)** | **Reported deep breathing (coverage %)** | **BCS/APVU/U (coverage %)¶** | **Mortality; n/N (%)** |
| --- | --- | --- | --- | --- | --- | --- | --- |
| Kabuchai, Kenya |  |  |  |  |  |  |  |
| 2019-21 | 164 (A) | 96.3 | 98.8 | 98.8 | 98.2 | AVPU (98.8) | 4/164 (2.4) |
| Kanduyi, Kenya |  |  |  |  |  |  |  |
| 2019-21 | 740 (A) | 97.0 | 98.9 | 99.1 | 98.9 | AVPU (99.1) | 5/739 (0.7) |
| Matayos, Kenya |  |  |  |  |  |  |  |
| 2019-21 | 652 (A) | 67.6 | 100 | 100 | 97.2 | AVPU (97.7) | 17/652 (2.6) |
| Teso South, Kenya |  |  |  |  |  |  |  |
| 2019-21 | 343 (A) | 74.6 | 100 | 100 | 96.5 | AVPU (98.5) | 8/343 (2.3) |
| Rachuonyo North, Kenya |  |  |  |  |  |  |  |
| 2019-21 | 23 (A) | 95.0 | 100 | 100 | 100 | AVPU (100) | 4/23 (17.4) |
| Rangwe, Kenya |  |  |  |  |  |  |  |
| 2019-21 | 20 (A) | 90.0 | 100 | 100 | 100 | AVPU (100) | 1/19 (5.3) |
| Suba North, Kenya |  |  |  |  |  |  |  |
| 2019-21 | 26 (A) | 100 | 100 | 100 | 100 | AVPU (100) | 1/26 (3.8) |
| Lurambi, Kenya |  |  |  |  |  |  |  |
| 2019-21 | 262 (A) | 74.8 | 100 | 100 | 100 | AVPU (100) | 6/262 (2.3) |
| Shinyalu, Kenya |  |  |  |  |  |  |  |
| 2019-21 | 64 (A) | 85.9 | 100 | 100 | 100 | AVPU (98.4) | 2/64 (3.1) |
| Kilifi North, Kenya |  |  |  |  |  |  |  |
| 2006-10 | 211 (A) | 97.2 | 99.5 | 100 | 99.5 | BCS (99.5) | 7/211 (3.3) |
| 2011-16 | 81 (A) | 98.8 | 100 | 100 | 98.8 | BCS (98.8) | 3/79 (3.8) |
| 2017-20 | 67 (A0 | 98.5 | 100 | 100 | 100 | BCS (100) | 3/66 (4.5) |
| Kilifi South, Kenya |  |  |  |  |  |  |  |
| 2006-10 | 723 (A) | 97.8 | 99.9 | 100 | 99.6 | BCS (99.2) | 16/719 (2.2) |
| 2011-16 | 926 (A) | 98.6 | 100 | 100 | 100 | BCS (100) | 31/915 (3.4) |
| 2017-20 | 325 (A) | 99.7 | 99.7 | 100 | 100 | BCS (100) | 13/323 (4) |
| Kisumu East, Kenya |  |  |  |  |  |  |  |
| 2015-18 | 274 (A) | 60.2 | 100 | 100 | 97.8 | AVPU (95.6) | 4/274 (1.5) |
| Kisumu West, Kenya |  |  |  |  |  |  |  |
| 2015-18 | 236 (A) | 57.6 | 99.6 | 99.6 | 99.2 | AVPU (95.3) | 4/236 (1.7) |
| Kisumu West, Kenya |  |  |  |  |  |  |  |
| 2019-21 | 157 (A) | 93.6 | 100 | 100 | 100 | AVPU (100) | 7/157 (4.5) |
| Kisumu Nyando, Kenya |  |  |  |  |  |  |  |
| 2019-21 | 105 (A) | 95.2 | 100 | 100 | 100 | AVPU (100) | 4/105 (3.8) |
| Kuria East & West, Kenya |  |  |  |  |  |  |  |
| 2019-21 | 43 (A) | 88.4 | 100 | 100 | 79.1 | AVPU (79.1) | 1/43 (2.3) |

**Table S2 continued**

| **Site, dates** | **No. malaria admissions (missing age) definition** | **Hb**  **(coverage %)** | **Reported transfusion ordered (coverage %)** | **SMA† (coverage %)** | **Reported deep breathing (coverage %)** | **BCS/APVU/U (coverage %)¶** | **Mortality; n/N (%)** |
| --- | --- | --- | --- | --- | --- | --- | --- |
| Suna East & West, Kenya |  |  |  |  |  |  |  |
| 2019-21 | 145 (A) | 90.3 | 100 | 100 | 86.2 | AVPU (77.2) | 4/143 (2.8) |
| Alego-usonga, Kenya |  |  |  |  |  |  |  |
| 2010-14 | 1385 (B) | 98.6 | 89.7 | 99.9 | 0 | U (84) | 19/1363 (1.4) |
| Gem, Kenya |  |  |  |  |  |  |  |
| 2010-14 | 82 (B) | 100 | 98.8 | 100 | 0 | U (98.8) | 2/82 (2.4) |
| Rarieda, Kenya |  |  |  |  |  |  |  |
| 2007-10 | 1985 (A) | 14.9 | 0 | 14.9 | 0 | AVPU (93.6) | 9/1944 (0.5) |
| 2011-16 | 1077 (A) | 20.3 | 0 | 20.3 | 0 | AVPU (99.7) | 11/1049 (1.0) |
| 2017-19 | 1139 (A) | 34.6 | 0 | 34.6 | 0 | AVPU (100) | 1/1119 (0.1) |
| Cherangany, Kenya |  |  |  |  |  |  |  |
| 2018-20 | 147 (A) | 58.5 | 100 | 100 | 100 | AVPU (100) | 4/143 (2.8) |
| Kiminini, Kenya |  |  |  |  |  |  |  |
| 2018-20 | 162 (A) | 59.9 | 100 | 100 | 99.4 | AVPU (99.4) | 5/160 (3.1) |
| Kwanza, Kenya |  |  |  |  |  |  |  |
| 2018-20 | 183 (A) | 62.3 | 100 | 100 | 98.9 | AVPU (98.4) | 5/179 (2.8) |
| Saboti, Kenya |  |  |  |  |  |  |  |
| 2018-20 | 198 (A) | 65.7 | 100 | 100 | 100 | AVPU (99.5) | 8/198 (4.0) |
| Hamisi, Kenya |  |  |  |  |  |  |  |
| 2019-21 | 112 (A) | 85.7 | 100 | 100 | 100 | AVPU (100) | 1/112 (0.9) |
| Sabatia, Kenya |  |  |  |  |  |  |  |
| 2019-21 | 157 (A) | 86.6 | 100 | 100 | 100 | AVPU (100) | 4/157 (2.5) |
| Apac, Uganda |  |  |  |  |  |  |  |
| 2012-15 | 8281 (A) | 3.2 | 16.3 | 18.0 | 92.6 | U (90.4) | 50/7993 (0.6) |
| 2017-18 | 2426 (A) | 0 | 32.9 | 32.9 | 98.7 | U (97.5) | 14/2218 (0.6) |
| Arua, Uganda |  |  |  |  |  |  |  |
| 2017-18 | 2124 (A) | 5.4 | 46.9 | 47.8 | 99.8 | U (100) | 103/1780 (5.8) |
| Jinja, Uganda |  |  |  |  |  |  |  |
| 2012-13 | 1985 (A) | 52.0 | 41.4 | 66.9 | 100 | U (100) | 29/1492 (1.9) |
| 2017-20 | 2741 (A) | 9.3 | 27.5 | 33.5 | 98.2 | U (97.1) | 42/2052 (2.0) |
| Kabale, Uganda |  |  |  |  |  |  |  |
| 2012-15 | 173 (A) | 31.8 | 32.4 | 53.8 | 100 | U (100) | 6/167 (3.6) |
| 2017 | 13 (A) | 15.4 | 15.4 | 23.1 | 100 | U (100) | 0/13 (0) |
| Kambuga, Uganda |  |  |  |  |  |  |  |
| 2012-15 | 1009 (A) | 4.0 | 67.7 | 69.2 | 99.1 | U (99) | 9/989 (0.9) |

**Table S2 continued**

| **Site, dates** | **No. malaria admissions (missing age) definition** | **Hb**  **(coverage %)** | **Reported transfusion ordered (coverage %)** | **SMA† (coverage %)** | **Reported deep breathing (coverage %)** | **BCS/APVU/U (coverage %)¶** | **Mortality; n/N (%)** |
| --- | --- | --- | --- | --- | --- | --- | --- |
| Mubende, Uganda |  |  |  |  |  |  |  |
| 2012-15 | 5419 (A) | 24.1 | 28.1 | 43.8 | 99.9 | U (99.9) | 126/4890 (2.6) |
| 2017-20 | 2736 (A) | 9.2 | 37.4 | 38.0 | 95.2 | U (93.9) | 30/2453 (1.2) |
| Tororo, Uganda |  |  |  |  |  |  |  |
| 2012-14 | 6954 (A) | 16.9 | 30.8 | 37.9 | 96.5 | U (96.3) | 43/6175 (0.7) |
| 2015 | 992 (A) | 8.7 | 16.0 | 21.3 | 95.3 | U (94.3) | 10/906 (1.1) |
| 2016-19 | 2912 (A) | 3.1 | 40.5 | 41.1 | 92.0 | U (90.4) | 21/2491 (0.8) |
| Bagamoyo, Tanzania |  |  |  |  |  |  |  |
| 2006-10 | 911 (A) | 0 | 100 | 100 | 0 | BCS (100) | 31/923 (3.4) |
| Handeni, Tanzania** |  |  |  |  |  |  |  |
| 2006-07 | 240 (B) | 100 | 100 | 100 | 100 | BCS (98.8) | 18/240 (7.5) |
| Mkinga, Tanzania** |  |  |  |  |  |  |  |
| 2006-07 | 91 (B) | 100 | 100 | 100 | 100 | BCS (100) | 5/91 (5.5) |
| Muheza, Tanzania** |  |  |  |  |  |  |  |
| 2006-07 | 1463 (B) | 100 | 100 | 100 | 100 | BCS (99.5) | 25/1463 (1.7) |

** In 3 time-site periods in Tanzania, data was available for children up to 13 years and not 14 years like the rest of the time-site periods

1. Malaria was defined as a primary discharge diagnosis with a confirmed positive slide/RDT but excluding major underlying conditions which included HIV, TB, Sickle Cell Disease, malignancies, epilepsy, measles or poisoning
2. Malaria was defined as a primary discharge diagnosis with a confirmed positive slide/RDT but with no clear exclusion of major underlying conditions

¶Cerebral malaria was defined as Blantyre Coma Score (BCS) of less than 3, if unconsciousness was observed or a score U in the AVPU (Alert, Voice, Pain, Unresponsive) scale

† Severe malaria anaemia (SMA) was defined as haemoglobin (Hb) < 5g/dl or using reported transfusion ordered where Hb was missing
