# Supplementary material for: Malaria hospitalisation in East Africa: age, phenotype and transmission intensity
Source: BMC Med. 2022 Jan 27;20:28. doi: 10.1186/s12916-021-02224-w (PMC8793189; doi:10.1186/s12916-021-02224-w)
Supplement: Supplementary file 3 — Additional file 3. Supplementary methods. [file 12916_2021_2224_MOESM3_ESM.docx]

**Malaria hospitalisation in East Africa: age, phenotype and transmission intensity**

Alice Kamau, Robert S Paton, Samuel Akech, Arthur Mpimbaza, Cynthia Khazenzi, Morris Ogweru, Eda Mumo, Victor A Alegana, Ambrose Agweyu, Neema Mturi, Shebe Mohammed, Godfrey Bigogo, Allan Audi, James Kapisi, Asadu Sserwanga, Jane F Namuganga, Simon Kariuki, Nancy A Otieno, Bryan O Nyawanda, Ally Olotu, Athuman Thabit, Nayha Salim, Salim Abdulla, Amina F Mohamed, George Mtove, Hugh Reyburn, Sunetra Gupta, José Lourenço, Philip Bejon, Robert W Snow

**Additional file 3: Supplementary methods**

We aimed to model how the average age of all-cause malaria admissions changed with parasite rate, while accounting for confounding inter-site factors intrinsic to the dataset. The age distribution (*A*) of cases admitted each hospital site-period (*H*) was modelled as a Gamma distribution with shape $\theta$ and rate *R*:

$$A \sim\Gamma\left( \theta,R^{H} \right)$$

The rate parameter is written as follows, where a log-linear function models the change in the distribution mean across hospital site-periods and with parasite rate (*PR^H,Y^*):

$$R^{H,Y}= \frac{\theta}{f(H,Y,S)}$$

$$\log\left[ f\left( H,Y,{PR}^{H,Y} \right) \right]=\alpha+\varphi^{H}+\beta{\times PR}^{H}$$

$$\varphi^{H}\sim N\left( 0,\sigma\right)$$

The intercept, *α*, is augmented by a random effect capturing hospital site-period specific deviations from the predicted mean age of admission were modelled with standard deviation *σ*. The effect of parasite rate was described by the coefficient β.

In a second Gamma-distributed GLMM, we considered differences in the age distribution of admissions for specific phenotypes (SMA, CM and RD). Records were filtered for admissions with a diagnosis of one or more of these phenotypes. Again, the mean of the distribution was modelled as a log-linear function of *Pf*PR_2-10_ and random effect terms modelled phenotype-specific site-time deviations. We explored three hypotheses for phenotype-specific age distributions. In the first, null, model, all cases of particular phenotypes were assumed to follow the same age distribution and respond to changes in parasite prevalence in the same way. In a second model, it was assumed that the age distribution of each phenotype had a different average age of admission but responded in the same way to changes in *Pf*PR_2-10_. A third model assumed that not only was the age distribution of phenotypes on average different, but that each phenotype also responded differently to changes in parasite prevalence. We compared these three models using the widely applicable information criterion, WAIC.
